# Establishment and application of multiplex real-time PCR for simultaneous detection of four viruses associated with porcine reproductive failure

**DOI:** 10.3389/fmicb.2023.1092273

**Published:** 2023-02-09

**Authors:** Yuan Chen, Shile Luo, Jianmei Tan, Luhua Zhang, Shengwu Qiu, Zhiyou Hao, Naidong Wang, Zhibang Deng, Aibing Wang, Qing Yang, Yi Yang, Changjian Wang, Yang Zhan

**Affiliations:** ^1^Provincial Key Laboratory of Protein Engineering in Animal Vaccines, Research Center of Reverse Vaccinology (RCRV), and Laboratory of Functional Proteomics (LFP), College of Veterinary Medicine, Hunan Agricultural University, Changsha, Hunan, China; ^2^Animal Disease Prevention and Control Center of Yongzhou, Yongzhou, Hunan, China; ^3^Animal Disease Prevention and Control Center of Hunan Province, Changsha, Hunan, China

**Keywords:** porcine circovirus type 2, porcine circovirus type 3, porcine parvovirus, pseudorabies virus, multiplex real-time PCR

## Abstract

Many pathogens cause reproductive failure in sows suffering a broad spectrum of sequelae, including abortions, stillbirth, mummification, embryonic death, and infertility. Although various detection methods, such as polymerase chain reaction (PCR) and real-time PCR, have been widely used for molecular diagnosis, mainly for a single pathogen. In this study, we developed a multiplex real-time PCR method for the simultaneous detection of porcine circovirus type 2 (PCV2), porcine circovirus type 3 (PCV3), porcine parvovirus (PPV) and pseudorabies virus (PRV) associated with porcine reproductive failure. The *R*^2^ values for the standard curve of multiplex real-time PCR of PCV2, PCV3, PPV, and PRV reached to 0.996, 0.997, 0.996, and 0.998, respectively. Importantly, the limit of detection (LoD) of PCV2, PCV3, PPV, and PRV, were 1, 10, 10, 10 copies/reaction, respectively. Meanwhile, specificity test results indicated that multiplex real-time PCR for simultaneous detection is specific for these four target pathogens and does not react with other pathogens, such as classical swine fever virus, porcine reproductive and respiratory syndrome virus, and porcine epidemic diarrhea virus. Besides, this method had good repeatability with coefficients of variation of intra- and inter-assay less than 2%. Finally, this approach was further evaluated by 315 clinical samples for its practicality in the field. The positive rates of PCV2, PCV3, PPV, and PRV were 66.67% (210/315), 8.57% (27/315), 8.89% (28/315), and 4.13% (13/315), respectively. The overall co-infection rates of two or more pathogens were 13.65% (43/315). Therefore, this multiplex real-time PCR provides an accurate and sensitive method for the identification of those four underlying DNA viruses among potential pathogenic agents, allowing it to be applied in diagnostics, surveillance, and epidemiology.

## Introduction

Some viruses causing reproductive failures in wild boar and domestic swine have posed significant economic threat to the swine industry (Pacini et al., 2021). Swine productions are significantly affected by the four main pathogenic DNA viruses, such as porcine circovirus type 2 (PCV2), porcine circovirus type 3 (PCV3), porcine parvovirus (PPV), and pseudorabies virus (PRV) (Díaz et al., 2021). PCV2 and PCV3 are non-enveloped single-stranded DNA viruses and the members of genus *Circovirus* of the *Circoviridae* family, and the two PCVs have a similar genomic structure: the viral genome contains two major open reading frames (ORFs) oriented in opposite directions, ORF1 and ORF2, which encode replication-related proteins and the capsid protein, respectively (Turlewicz-Podbielska et al., 2022). Furthermore, co-infections of PCV2 and PCV3 were found in swine herds, which can cause similar clinical manifestations including reproductive failure, porcine dermatitis, nephropathy syndrome and respiratory disease (Xu et al., 2021; Sirisereewan et al., 2022). PPV is also a non-enveloped single-stranded DNA virus, and its genome contains two ORFs: ORF1 encodes nonstructural proteins of NS1 and NS2, while ORF2 encodes two structural proteins of VP1 and VP2 (Streck and Truyen, 2020). Among seven PPV types, PPV type 1 (PPV1) is a dominant etiological agent causing reproductive disorders in Chinese swine herds (Li et al., 2021). PRV is an enveloped double-stranded DNA virus, and belongs to the subfamily of *alpha-herpesvirus* in the family of *Herpesviridae* (Xu et al., 2020). PRV can cause neurological disorders and reproductive failure in swine, which is considered one of the most economically important viral swine diseases worldwide (Lv et al., 2020).

The four viruses with similar clinical manifestations including reproductive failure cause severe economic losses to the swine industry and world trade of pork products, however, differential diagnosis of diseases caused by these four viruses is difficult. Furthermore, co-infections with more than two pathogens of PCV2, PCV3, PPV, and PRV occur frequently in swine herds in China and elsewhere worldwide (Ma et al., 2021; Serena et al., 2021; Jia et al., 2022). Therefore, the prevention and control of these diseases caused by the four different viruses mainly depend on the accurate and rapid molecular diagnosis, and it is essential to develop a specific, high-sensitive, and rapid assay being capable of the simultaneous and differential detection of PCV2, PCV3, PPV, and PRV.

Among various detection methods, real-time PCR is an accurate, sensitive, and rapid method for the detection and quantification of the target genome, which is based on continuous measurements of the accumulation of fluorescent signals during the amplification reaction. However, conventional singular real-time PCR is time-consuming and costly to detect multiple pathogens in the same sample. With the development of this technology, most real-time PCR are based on the use of target-specific TaqMan probes, and multiplex real-time PCR can simultaneously detect more than one target gene with one reaction at one time (Hawkins and Guest, 2017; Zhu et al., 2022). In this study, primers and probes were designed based on the conserved fragments of the *cap* gene of PCV2, the *cap* gene of PCV3, the *NS1* gene of PPV and the *gE* gene of PRV, and a TaqMan probe-based multiplex real-time PCR method was successfully developed. The method showed high sensitivity and specificity, without cross-reactions with other tested swine pathogen genomes. Additionally, this approach provides an accurate identification of the pathogenic agents among the four underlying DNA viruses. This approach can thus be applied in diagnostics, surveillance, and epidemiology.

## Materials and methods

### Nucleic acids of viruses and clinical samples

Genomes (DNA and cDNA) of PCV2, PCV3, PPV, PRV, classical swine fever virus (CSFV), porcine reproductive and respiratory syndrome virus (PRRSV), and porcine epidemic diarrhea virus (PEDV) were all provided by the Animal Disease Prevention and Control Center of Hunan Province or the Provincial Key Laboratory of Protein Engineering in Animal Vaccines of Hunan Province. To test clinical samples from pig farms with reproductive disorders, DNA samples were extracted from tissues or blood by using the Tissue Genomic DNA Extraction Kit (Tiangen Biotech).

### Primers and TaqMan probes

**Figure 1:**
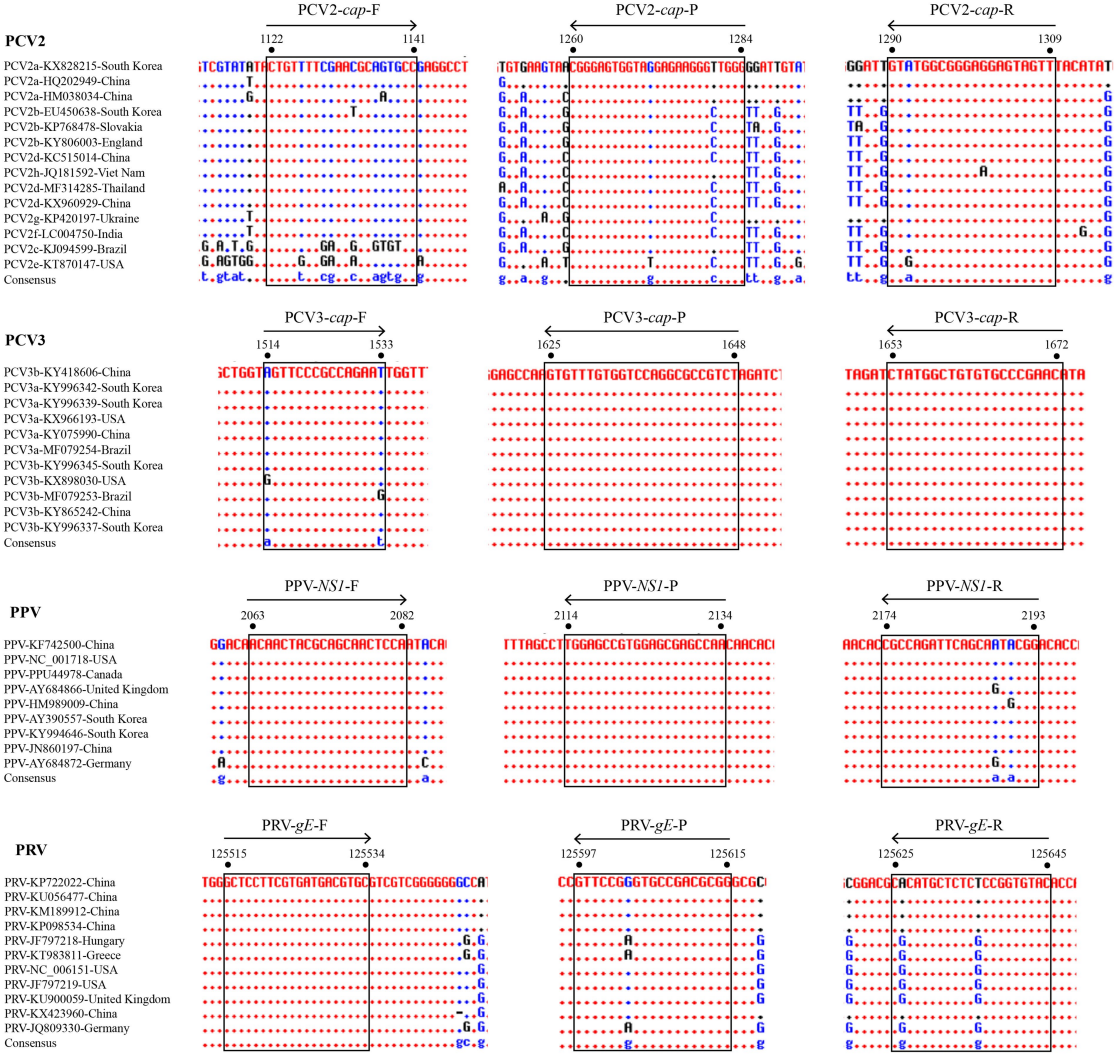
Alignments of coding sequences of reference viral strains deposited in GenBank. The locations of the primers/probes specific for PCV2 *cap* gene, PCV3 *cap* gene, PPV *NS1* gene, and PRV *gE* gene are shown. The positions of the partial nucleotide fragments are indicated by numbers.

For primers and probe design, different numbers of representative viral genomes (14 for PCV2, 11 for PCV3, 9 for PPV, and 11 for PRV) were obtained from GenBank, as reported and presented in genetic studies (Oh et al., 2017; Franzo and Segalés, 2018; Fux et al., 2018; He et al., 2019). Three distinct primer sets for each viral gene were designed using Primer Premier v5.0 based on the conserved region of reference strains, and further confirmed using the Blast tool available from the National Center for Biotechnology Information (NCBI). After these three sets of primers were tested by traditional PCR and then used with an appropriate probe to establish standard curves, the final primers/probes were determined after comparing the differences in specificity and sensitivity between the three sets of results. The locations of these final primers and probes were shown among different reference strains of PCV2, PCV3, PPV, and PRV (Figure 1). Detailed information of these primers and probes is listed in Table 1. For multiplex detection, the probe for the four viral genes were labeled with the different 5′-reporting dyes (FAM, VIC, ROX and Cy5) and the corresponding 3′-quenchers (MGB, BHQ1, BHQ2, and BHQ3). In addition, the sequences of the primers specific for three RNA viruses (CSFV, PRRSV, and PEDV) are shown in Supplementary Table S1.

**Table 1:** Primers and probes used in the multiplex real-time PCR assays.

Name	Sequence (5′- 3′)	Product size (bp)
PCV2-*cap*-F	CTGTTTTCGAACGCAGTGCC	188
PCV2-*cap*-R	AACTACTCCTCCCGCCATAC	
PCV2-*cap*-P	FAM-CCCAGCCCTTCTCCTACCACTCCCG-MGB	
PCV3-*cap*-F	CTGGTAGTTCCCGCCAGAAT	159
PCV3-*cap*-R	GTTCGGGCACACAGCCATAG	
PCV3-*cap*-P	VIC-AGACGGCGCCTGGACCACAAACAC-BHQ1	
PPV-*NS1*-F	ACAACTACGCAGCAACTCCA	131
PPV- *NS1*-R	CCGTATTGCTGAATCTGGCG	
PPV- *NS1-*P	ROX-TTGGCTCGCTCCACGGCTCCA-BHQ2	
PRV-*gE*-F	GCTCCTTCGTGATGACGTGC	131
PRV-*gE* -R	GTACACCGGAGAGAGCATGTG	
PRV-*gE* -P	Cy5-CCGCGTCGGCACCCGGAAC-BHQ3	

### Preparation of standard plasmid

**Figure 2:**
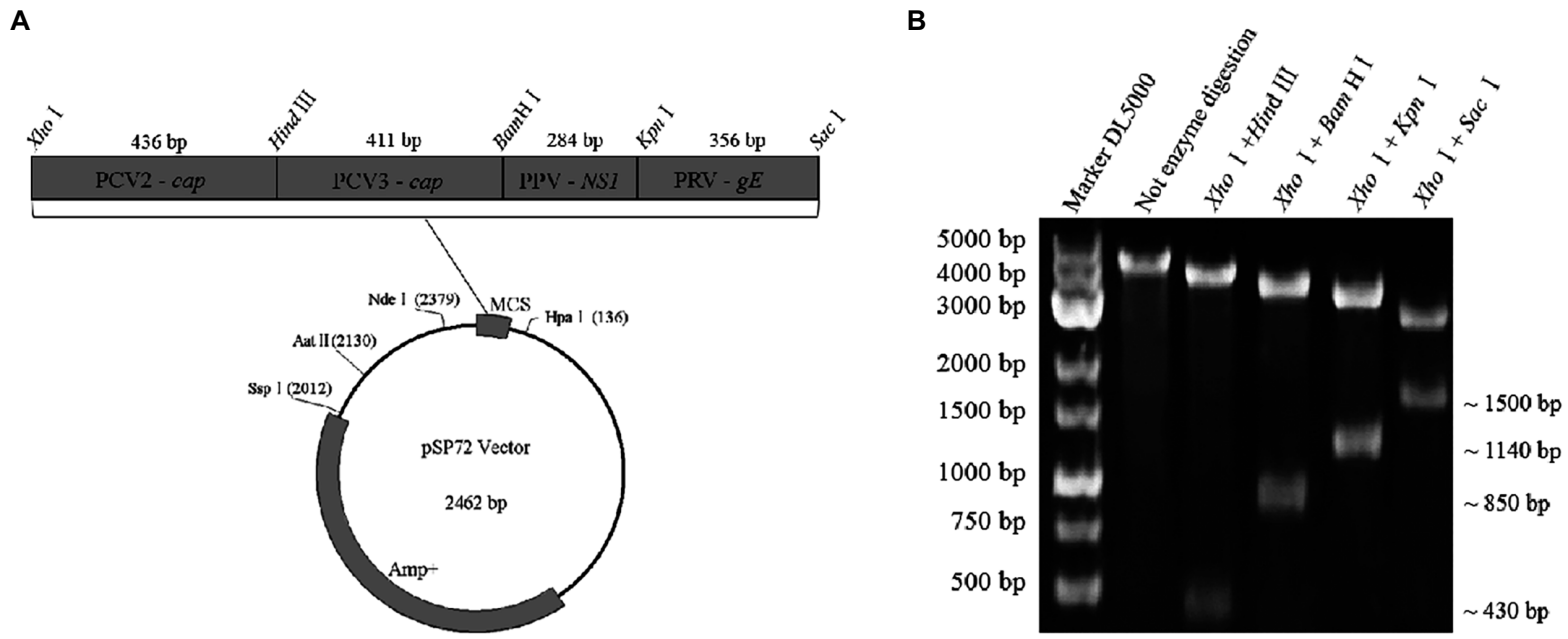
Construction and identification of a recombinant plasmid contains four genes in tandem. **(A)** Representative diagram of recombinant plasmid. **(B)** Restriction digestion assays of recombinant plasmid: ~430 bp in size specific for the *cap* gene of PCV2, ~850 bp in size specific for the two gene fragments (PCV2 *cap* and PCV3 *cap*) in tandem, ~1,140 bp in size specific for the three gene fragments (PCV2 *cap*, PCV3 *cap*, and PPV *NS1*) in tandem, ~1,500 bp in size specific for the four gene fragments (PCV2 *cap*, PCV3 *cap*, PPV *NS1*, and PRV *gE*) in tandem were identified by digestion with *Xho*I/*Hin*dIII, *Xho*I/*Bam*HI, *Xho*I/*Kpn*I and *Xho*I/*Sac*I, respectively.

Four gene fragments of PCV2, PCV3, PPV, and PRV, generated by PCR using their respective genomes as templates, were simultaneously inserted into a cloning vector of pSP72 (Promega) in tandem and served as a standard plasmid (pSP72-PCV2 *cap* & PCV3 *cap* & PPV *NS1* & PRV *gE*) in subsequent assays (Figure 2A). Briefly, the four gene fragments were digested with *Xho*I/*Hin*dIII, *Hin*dIII/*Bam*HI, *Bam*HI/*Kpn*I, and *Kpn*I/*Sac*I(Thermo Fisher Scientific), and then purified and ligated to the vector digested with *Xho*I/ *Sac*I. Finally, the ligation products were transformed into *Escherichia coli* DH5α competent cells (TransGen). The positive clones were cultured at 37°C for 20 h then extracted by Plasmid DNA Mini Kit (Omega) and confirmed by DNA sequencing. The resulting plasmids were quantified by ultraviolet absorbance at 260 nm and 280 nm using a NanoDrop spectrophotometer (Thermo Fisher, USA). According to the size of the standard plasmid template, the copy numbers of the purified plasmids were calculated based on the following formula: (A260 (ng/μL) × 10^−9^ × 6.02 × 10^23^) / (DNA length×650) = copies/μL. Then, 10-fold serial dilutions of the standard plasmid, with the same starting concentration of 10^9^ copies/μL, were performed to generate four standard curves for testing the multiplex real-time PCR.

### Establishment of multiplex real-time PCR assays

Multiplex real-time PCR was performed in a 20 μL reaction mixture containing 10 μL of 2 × AceQ real-time PCR Probe Master Mix (Vazyme), 0.4 μL of each primer (final concentration of 400 nM), 0.2 μL of each probe (final concentration of 200 nM), 1 μL of DNA template, and an appropriate amount of RNase-free water. The amplification parameters of the qPCR detection system were as follows: denaturation at 95°C for 5 min, followed by 45 cycles of denaturation at 95°C for 10 s, and annealing and extension at 60°C for 30 s. The acquisition of fluorescent signals was recorded at the end of each cycle. After the reaction, the system automatically determined the Ct values. The standard plasmid was 10-fold serially diluted from 10^8^ copies/μL to 10^1^ copies/μL and used as templates to construct the standard curves of the multiplex real-time PCR.

### Evaluation of specificity and sensitivity

To evaluate the specificity of primer and probe sets, cDNA of CSFV, PRRSV, and PEDV were confirmed *via* PCR with gene-specific primers serving as templates for amplification using the developed multiplex real-time PCR. For the sensitivity evaluation, the standard plasmid was serially diluted 10-fold and the final concentration ranged from 1 × 10^6^ copies/μL to 1 × 10^0^ copies/μL. These diluted plasmids were used as templates to determine the limit of detection (LoD) of the multiplex real-time PCR and each reaction was repeated triplicate in a test. To ensure the accuracy of the limit of LoD, 100 copies/μL, 10 copies/μL and 1 copy/μL of standard plasmid were tested repeatedly at 20 times, and the lowest concentration of the positive control that can be positively detected (60% replicates positive) was set as the LoD and labeled in the amplification curve. We judged the positive detection of the lowest concentration (100 copies/μL, 10 copies/μL and 1 copy/μL) according to the criteria of method and calculated the positive detection rate.

### Diagnostic performance of the multiplex real-time PCR

To evaluate the repeatability of the established multiplex real-time PCR, 10^6^, 10^4^ and 10^2^ copies/μL of the abovementioned standard plasmid were selected as templates to conduct the intra-assay and inter-assay tests. All reactions were repeated three times in one experiment.

### Detection of clinical samples by probe-based real-time PCR

A total of 315 clinical swine samples including lung, lymph nodes and blood, collected in Hunan Province, Southern China, were tested to detect the PCV2, PCV3, PPV, and PRV by the multiplex real-time PCR established in this study. In the comparative experiment, some pathogen-positive and pathogen-negative samples (n > 10) confirmed by the developed multiplex real-time PCR were selected. 100 pathogen-positive specimens (84 for PCV2, 26 for PCV3, 27 for PPV, 13 for PRV, including 43 co-infected samples, as shown in Supplementary Table S2) and 14 pathogen-negative specimens were selected and further analyzed by four singular real-time PCR. The primers and probes of singular real-time PCR were described from other laboratories (Song et al., 2010; Wang et al., 2017; Henriques et al., 2018; Tu et al., 2021). For each singular real-time PCR, a 20 μL reaction mixture containing 10 μL of 2 × AceQ real-time PCR Probe Master Mix (Vazyme), 0.4 μL of each primer (final concentration of 400 nM), 0.2 μL of probe (final concentration of 200 nM), 1 μL of DNA template, and an appropriate amount of RNase-free water. The amplification parameters of the qPCR detection system for singular real-time PCR were as follows: denaturation at 95°C for 5 min, followed by 45 cycles of denaturation at 95°C for 10 s, and annealing and extension at 60°C for 30 s. The acquisition of fluorescent signal (FAM) was recorded at the end of each cycle. After the reaction, the system automatically determined the Ct values. According to its application in sample detection in our laboratory, the criteria of singular real-time PCR for judging the outcome were as follows: A Ct value less than 40.0 cycles was considered positive, otherwise negative.

## Results

### Construction and validation of the standard plasmid

A standard plasmid containing the four gene fragments of PCV2 *cap*, PCV3 *cap*, PPV *NS1*, and PRV *gE* in tandem was successfully constructed and analyzed by restriction enzyme digestions assays. As expected, 4 gene fragments with distinct sizes were identified after restrictions (Figure 2B). Finally, the plasmid was further confirmed by double-strand DNA sequencing, and it was used as the standard plasmid for the subsequent assays.

### Establishment of the standard curve

**Figure 3:**
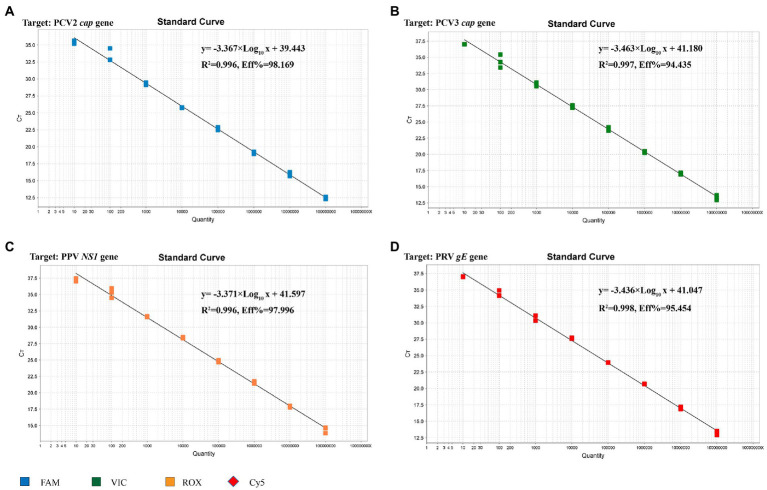
Standard curve for multiplex real-time PCR assay. **(A)** The standard curve for the PCV2 *cap* gene. **(B)** The standard curve for the PCV3 *cap* gene. **(C)** The standard curve for the PPV *NS1* gene. **(D)** The standard curve for the PRV *gE* gene.

The standard plasmid was subjected to 10-fold serial dilutions to establish the standard curve, and 8 standard samples (10^8^ ~ 10^1^ copies/μL) were selected as templates to establish a standard curve for the multiplex real-time PCR. As shown in Figure 3, the corresponding correlation coefficient (R^2^), slope of the equation and amplification efficiency (E) were as follows: 0.996, −3.367, and 98.169%, respectively, for PCV2; 0.997, −3.463, and 94.435%, respectively, for PCV3; 0.996, −3.371, and 97.996%, respectively, for PPV; 0.998, −3.436, and 95.454%, respectively, for PRV. Both R^2^ and E indicated a good linear relationship between the initial templates and Ct values. According to the intercepts of the standard curve, the criteria for judging the outcome were as follows: A Ct value less than 40.0 cycles was considered positive, otherwise negative.

### Specificity evaluation

**Figure 4:**
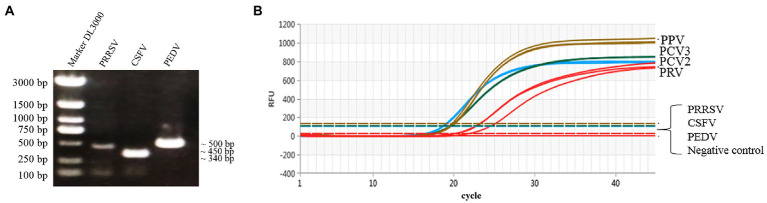
Specific amplification curves by multiplex real-time PCR assay. **(A)** Agarose gel electrophoresis of viral replicons (PRRSV, CSFV, and PEDV) amplified by PCR. Lane 1 represents the 3,000-bp marker, Lanes 2–4 represent PRRSV (451 bp), CSFV (343 bp) and PEDV (503 bp), respectively. **(B)** Four fluorescent signals were monitored by multiplex real-time PCR, and DNA of PCV2, PCV3, PPV, and PRV were used as a positive control. No fluorescent signal was observed when other viral cDNAs were used as templates. Note: The graph type was set as in linear phase to simultaneously display the four different fluorescent signals (FAM, VIC, ROX, and Cy5) with distinct signal intensities.

The genomic DNA or cDNA of different porcine viruses (PCV2, PCV3, PPV, PRV, CSFV, PRRSV, and PEDV) were used as templates of the multiplex real-time PCR, and cDNA of CSFV, PRRSV, and PEDV were confirmed *via* PCR with gene-specific primers (Figure 4A). As a result, only PCV2, PCV3, PPV, and PRV showed amplification curves, while no detectable fluorescent signal was observed among the templates derived from CSFV, PRRSV and PEDV (Figure 4B). Thus, the established multiplex real-time PCR is specific for the detection of the four viruses (PCV2, PCV3, PPV, and PRV) herein investigated and has no cross-reactivity with the other 3 swine pathogens.

### Sensitivity evaluation

**Figure 5:**
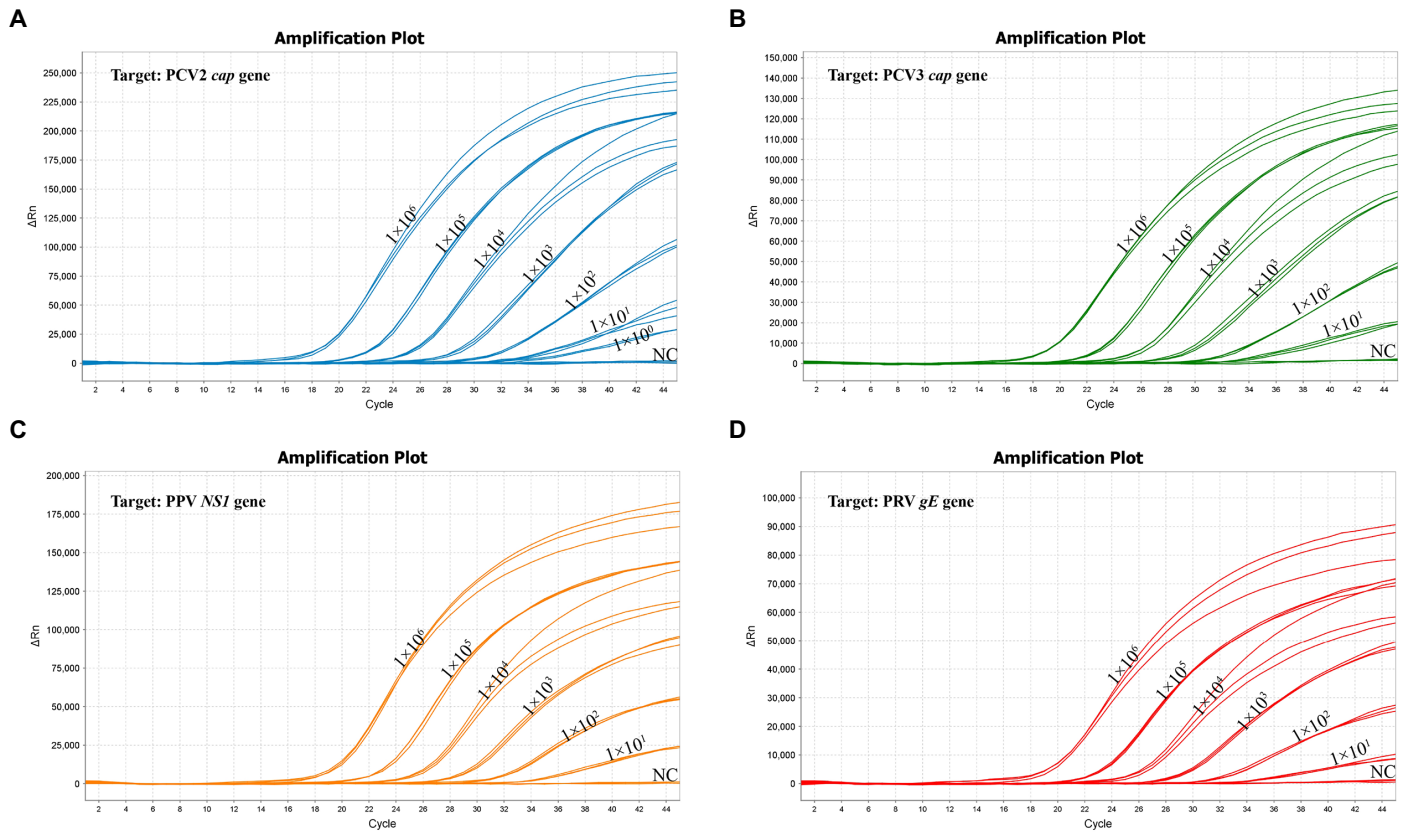
The sensitivity of multiplex real-time PCR assay. **(A)** The sensitivity for the PCV2 *cap* gene. **(B)** The sensitivity for the PCV3 *cap* gene. **(C)** The sensitivity for the PPV *NS1* gene. **(D)** The sensitivity for the PRV *gE* gene.

The standard plasmid was serial diluted 10-fold from 1 × 10^6^ copies/μL to 1 × 10^0^ copies/μL to determine the LoD of the multiplex real-time PCR. The Lod of PCV2 was 1 copy/μL, and the Lod of all other 3 viruses (PCV3, PPV and PRV) were 10 copies/μL (Figure 5; Supplementary Table S3), highlighting the high sensitivity of the multiplex real-time PCR.

### Repeatability evaluation

Three concentrations of 1 × 10^6^, 1 × 10^4^ and 1 × 10^2^ copies/μL of standard plasmids were used to evaluate the reproducibility of the multiplex real-time PCR. As a result, the coefficients of variation of the intra- and inter-assay ranged from 0.03% to 1.50% and 0.16% to 1.82%, respectively (Table 2), indicating good repeatability of the assay.

**Table 2:** Repeatability and reproducibility evaluation of the multiplex real-time PCR.

Target	Concentration (copies/μL)	Intra-assay			Inter-assay		
		Mean Ct value	SD	CV(%)	Mean Ct value	SD	CV(%)
PCV2	10^2^	32.62	0.13	0.39	32.70	0.60	1.82
	10^4^	26.11	0.11	0.43	26.21	0.30	1.13
	10^6^	19.28	0.14	0.74	19.22	0.03	0.16
PCV3	10^2^	34.36	0.01	0.03	34.14	0.55	1.60
	10^4^	27.34	0.18	0.64	27.37	0.10	0.38
	10^6^	20.40	0.04	0.21	20.49	0.15	0.73
PPV	10^2^	34.26	0.51	1.50	34.80	0.21	0.61
	10^4^	28.36	0.08	0.29	28.26	0.12	0.41
	10^6^	21.62	0.10	0.46	21.66	0.15	0.71
PRV	10^2^	34.31	0.34	1.00	34.60	0.38	1.11
	10^4^	27.37	0.11	0.42	27.28	0.25	0.92
	10^6^	20.91	0.04	0.21	20.87	0.10	0.48

SD, standard deviation, CV, coefficient of variation. and mean Ct value was calculated from three independent wells for duplex real-time PCR runs.

### The detection results of clinical samples

A total of 315 clinical swine samples were collected from 14 regions in Hunan Province, Southern China, and detected using the developed multiplex real-time PCR to evaluate its practicality in the field. As a result (Table 3), the positive rates of PCV2, PCV3, PPV, and PRV were 66.67% (210/315), 8.57% (27/315), 8.89% (28/315), and 4.13% (13/315), respectively. Furthermore, the positive rates of co-infections with two or more distinct viruses were 13.65% (43/315; Table 3), and the Ct values obtained from the four fluorescent channels (FAM, VIC, ROX, and Cy5) from these 43 samples were shown (Supplementary Table S2), indicating that this method can really detect multiple viruses simultaneously.

**Table 3:** Detection results of clinical samples by the multiplex real-time PCR.

Region	Number	Number of positive samples				
		PCV2	PCV3	PPV	PRV	Co-infections
Changde	29	27	2	8	7	14
Yueyang	15	9	2	0	0	0
Yiyang	63	50	6	4	0	10
Hengyang	17	13	4	4	1	6
Zhuzhou	20	6	3	2	1	3
Chenzhou	11	4	0	0	0	0
Loudi	10	6	0	0	0	0
Huaihua	44	18	1	2	0	2
Yongzhou	7	4	1	2	0	1
Changsha	23	12	4	2	2	3
Xiangtang	12	4	1	2	2	2
Xiangxi	7	4	1	1	0	1
Liuyang	53	50	2	1	0	1
Shaoyang	4	3	0	0	0	0
Total	315	210	27	28	13	43
Positive rate (%)		66.67	8.57	8.89	4.13	13.65

Co-infections means infection with two or more detected pathogens (PCV2, PCV3, PPV, and PRV).

Among the verified DNA samples, 100 pathogen-positive specimens (including above 43 co-infected samples and 57 singular infected samples) and 14 pathogen-negative specimens were also detected by the singular real-time PCR. As shown in Table 4, the positive rates of PCV2, PCV3, PPV, and PRV were 68.42% (78/114), 21.05% (24/114), 20.18% (23/114), and 9.65% (11/114), respectively, and the agreement rate of the developed multiplex real-time PCR and the singular real-time PCR was 94.01% (PCV2), 95.47% (PCV3), 90.82% (PPV) and 91.14% (PRV), respectively. In addition, no sample among 14 pathogen-negative specimens was tested positive for these pathogens (PCV2, PCV3, PPV, and PRV) by the singular real-time PCR.

**Table 4:** Comparative results of clinical samples tested with both multiplex real-time PCR and singular real-time PCR.

Detection method	Clinical samples	Number of positive samples			
		PCV2	PCV3	PPV	PRV
Multiplex real-time PCR	43 co-infected samples	43/43	15/43	23/43	12/43
	57 singular infected samples	41/57	11/57	4/57	1/57
	14 negative samples	0/14	0/14	0/14	0/14
	Totally 114 samples	84/114	26/114	27/114	13/114
Singular real-time PCR	Above 114 samples	78/114	24/114	23/114	11/114
Agreements		94.01%	95.47%	90.82%	91.14%

## Discussion

International travel and large-scale logistics significantly increase the prevalence and spread of animal pathogens, such as PCV2, PPV, and PRV, leading not only to animal infections, but also the substantial economic loss to the pig industry (VanderWaal and Deen, 2018). PCV2, PCV3, PPV, and PRV are all DNA viruses causing reproductive failures that have seriously negative effects on the swine industry worldwide. These viruses are still prevalent in many countries and areas, and co-infections with each other are common in some pig herds (Zhao et al., 2019; Ma et al., 2020; Zhou et al., 2020; Kang et al., 2022; Kim et al., 2022). Because pigs infected by these viruses sometimes show similar clinical symptoms, it is difficult to identify the key causative agent(s) only by clinical observations (Zhang et al., 2019; Vargas-Bermúdez et al., 2021; Li et al., 2022). Multiplex real-time PCR uses gene-specific primers and probes to amplify several target genes simultaneously in a single tube. Therefore, the assay is rapid, sensitive, accurate, and time-saving, and it has been widely used to detect various viruses in veterinary laboratories (Scherrer and Stephan, 2021; Liu et al., 2022). Thus, it is pertinent to develop a reliable method for the simultaneous detection of PCV2, PCV3, PPV, and PRV in laboratory and accurate diagnosis for the diseases associated with the four viruses in the field.

In this study, four pairs of viral gene-specific primers and corresponding probes (Table 1; Figure 1) were designed for the multiplex real-time PCR, which can simultaneously detect the presences of the four genes *via* different fluorescent reporters in one tube. Furthermore, instead of using a mixture of four standard plasmids, we inserted the four gene fragments into the same vector in tandem to generate one standard plasmid for use in the multiplex real-time PCR system (Figure 2). The advantages of using one standard plasmid harboring four viral gene fragments in this assay will decrease the cost of preparing a standard plasmid compared to using four standard plasmids. Besides, this approach may decrease system errors caused by the need to add four distinct plasmids. The sensitivity assay demonstrated that this method could detect less than 10 copies of target genes in our standard plasmid harboring the four genes (Figure 5; Supplementary Table S3), and the standard curve plots showed a strong linear correlation between the Ct value and the standard copy numbers (Figure 3). Based on the specificity of the multiplex probe-based real-time PCR, this assay could accurately detect PCV2, PCV3, PPV, and PRV without cross-reactions with other porcine RNA viruses (CSFV, PRRSV, and PEDV; Figure 4). Furthermore, the developed multiplex real-time PCR was experimentally verified with confirmed ASFV-positive samples, and the results demonstrated good specificity of the assay, which could specifically detect four viruses herein investigated (data not shown). Meanwhile, more herein investigated pathogen-positive samples need to be tested to confirm the developed multiplex real-time PCR have a broad utility, and further studies are warranted.

In addition, the assay was further employed to detect 315 clinical samples to verify its practicality and usefulness in the clinical samples. Our results demonstrate that 210 (66.67%), 27 (8.57%), 28 (8.89%), and 13 (4.13%) samples were positive for PCV2, PCV3, PPV, and PRV, respectively (Table 3). This suggests that PCV2, PCV3, PPV, and PRV were still prevalent in Hunan province, Southern China. Furthermore, co-infections with two or more pathogens among PCV2, PCV3, PPV, and PRV are also common, which may elevate immunosuppression and inflammation, and thus increase the possibility of secondary infection by other pathogen(s), and further exacerbate these diseases (Vargas-Bermúdez et al., 2021; Wu et al., 2021; Li et al., 2022). The detection results of the clinical samples showed that co-infections with 2 or more pathogens of PCV2, PCV3, PPV, and PRV still existed in 43 (13.65%) samples (Table 3; Supplementary Table S2) in Hunan province. At the same time, 114 out of 315 clinical samples were selected and detected by using the singular real-time PCR. The results indicated that the agreement rate of the developed multiplex real-time PCR and the singular real-time PCR were more than 90%, and the multiplex real-time PCR was more sensitive than singular real-time PCR (Table 4).

In conclusion, a multiplex real-time PCR was developed for the simultaneous and differential detection of PCV2, PCV3, PPV, and PRV to rapidly and accurately detect PCV2, PCV3, PPV, and PRV from clinical samples, this novel method could be used as a more convenient tool for the accurate diagnosis and epidemiological investigation of these viruses.

## Data availability statement

The original contributions presented in the study are included in the article/Supplementary material, further inquiries can be directed to the corresponding authors.

## Ethics statement

The animal study was reviewed and approved by the Ethics Committee for Biomedical Research of Hunan Agricultural University, Changsha, China, following national legislation regarding animal welfare. Written informed consent was obtained from the owners for the participation of their animals in this study.

## Author contributions

YZ, CW, ZD, and YY conceived and designed the study. YC, SQ, JT, LZ, SL, and ZH performed the experiments and analyzed data. YC, SQ, and YZ wrote the paper, NW, QY, and AW revised the manuscript. All authors contributed to the article and approved the submitted version.

## Funding

This work was supported by National Natural Science Foundation of China (32172844 and 32202790), Hunan Provincial Natural Science Foundation of China (2022JJ30298 and 2022JJ40183), Natural Science Foundation of Changsha City (kq2202231), the Young Talents Science and Technology Project in Hunan Province (2022RC1046), and Hunan Province Technology Breakthrough Project of 2021 for the open competition mechanism to select the best candidates (grant number: 2021NK1030).

## Conflict of interest

The authors declare that the research was conducted in the absence of any commercial or financial relationships that could be construed as a potential conflict of interest.

## Publisher’s note

All claims expressed in this article are solely those of the authors and do not necessarily represent those of their affiliated organizations, or those of the publisher, the editors and the reviewers. Any product that may be evaluated in this article, or claim that may be made by its manufacturer, is not guaranteed or endorsed by the publisher.

## References

[ref1] Díaz C. Celer V. Frébort I. (2021). The Main DNA viruses significantly affecting pig livestock. J. Vet. Res. 65, 15–25. doi: 10.2478/jvetres-2021-0001, PMID: 33817391 PMC8009578

[ref2] Franzo G. Segalés J. (2018). Porcine circovirus 2 (PCV-2) genotype update and proposal of a new genotyping methodology. PLoS. One 13:e0208585. doi: 10.1371/journal.pone.0208585, PMID: 30521609 PMC6283538

[ref3] Fux R. Söckler C. Link E. K. Renken C. Krejci R. Sutter G.. (2018). Full genome characterization of porcine circovirus type 3 isolates reveals the existence of two distinct groups of virus strains. Virol. J. 15:25. doi: 10.1186/s12985-018-0929-3, PMID: 29378597 PMC5789634

[ref4] Hawkins S. F. C. Guest P. C. (2017). Multiplex analyses using real-time quantitative PCR. Methods Mol. Biol. 1546, 125–133. doi: 10.1007/978-1-4939-6730-8_8 27896761

[ref5] He W. Auclert L. Z. Zhai X. Wong G. Zhang C. Zhu H.. (2019). Interspecies transmission, genetic diversity, and evolutionary dynamics of Pseudorabies virus. J. Infect. Dis. 219, 1705–1715. doi: 10.1093/infdis/jiy731, PMID: 30590733

[ref6] Henriques A. M. Duarte M. Barros S. C. Fagulha T. Ramos F. Luís T.. (2018). Development and validation of a real-time PCR for the detection and quantification of porcine circovirus type 2. Virus 29, 355–361. doi: 10.1007/s13337-018-0476-y, PMID: 30159371 PMC6111954

[ref7] Jia Y. Zhu Q. Xu T. Chen X. Li H. Ma M.. (2022). Detection and genetic characteristics of porcine circovirus type 2 and 3 in Henan province of China. Mol. Cell. Probes 61:101790. doi: 10.1016/j.mcp.2022.101790, PMID: 35051595

[ref8] Kang L. Wahaab A. Shi K. Mustafa B. E. Zhang Y. Zhang J.. (2022). Molecular epidemic characteristics and genetic evolution of porcine Circovirus type 2 (PCV2) in swine herds of Shanghai, China. Viruses 14:289. doi: 10.3390/v14020289, PMID: 35215883 PMC8879946

[ref9] Kim S. C. Kim J. H. Kim J. Y. Park G. S. Jeong C. G. Kim W. I. (2022). Prevalence of porcine parvovirus 1 through 7 (PPV1-PPV7) and co-factor association with PCV2 and PRRSV in Korea. BMC Vet. Res. 18:133. doi: 10.1186/s12917-022-03236-1, PMID: 35395853 PMC8994367

[ref10] Li X. Chen S. Zhang L. Niu G. Zhang X. Yang L.. (2022). Coinfection of porcine Circovirus 2 and Pseudorabies virus enhances immunosuppression and inflammation through NF-κB, JAK/STAT, MAPK, and NLRP3 pathways. Int. J. Mol. Sci. 23:4469. doi: 10.3390/ijms23084469 35457287 PMC9029761

[ref11] Li J. Xiao Y. Qiu M. Li X. Li S. Lin H.. (2021). A systematic investigation unveils high Coinfection status of porcine parvovirus types 1 through 7 in China from 2016 to 2020. Microbiol. Spectr. 9:e0129421. doi: 10.1128/Spectrum.01294-21, PMID: 34851175 PMC8635132

[ref12] Liu H. Shi K. Zhao J. Yin Y. Chen Y. Si H.. (2022). Development of a one-step multiplex qRT-PCR assay for the detection of African swine fever virus, classical swine fever virus and atypical porcine pestivirus. BMC Vet. Res. 18:43. doi: 10.1186/s12917-022-03144-4, PMID: 35042532 PMC8764768

[ref13] Lv L. Cao M. Bai J. Jin L. Wang X. Gao Y.. (2020). PRV-encoded UL13 protein kinase acts as an antagonist of innate immunity by targeting IRF3-signaling pathways. Vet. Microbiol. 250:108860. doi: 10.1016/j.vetmic.2020.108860, PMID: 33045632

[ref14] Ma Z. Han Z. Liu Z. Meng F. Wang H. Cao L.. (2020). Epidemiological investigation of porcine pseudorabies virus and its coinfection rate in Shandong Province in China from 2015 to 2018. J. Vet. Sci. 21:e36. doi: 10.4142/jvs.2020.21.e36, PMID: 32476312 PMC7263908

[ref15] Ma Z. Liu M. Liu Z. Meng F. Wang H. Cao L.. (2021). Epidemiological investigation of porcine circovirus type 2 and its coinfection rate in Shandong province in China from 2015 to 2018. BMC Vet. Res. 17:17. doi: 10.1186/s12917-020-02718-4, PMID: 33413367 PMC7792206

[ref16] Oh W. T. Kim R. Y. Nguyen V. G. Chung H. C. Park B. K. (2017). Perspectives on the evolution of porcine parvovirus. Viruses 9:196. doi: 10.3390/v9080196, PMID: 28933737 PMC5580453

[ref17] Pacini M. I. Forzan M. Cilia G. Bertelloni F. Fratini F. Mazzei M. (2021). Detection and characterization of viral pathogens associated with reproductive failure in wild boars in Central Italy. Animals (Basel) 11:304. doi: 10.3390/ani11020304 33504030 PMC7911021

[ref18] Scherrer S. Stephan R. (2021). Novel multiplex TaqMan assay for differentiation of the four major pathogenic Brachyspira species in swine. Microbiology 10:e1169. doi: 10.1002/mbo3.1169, PMID: 33650802 PMC7887428

[ref19] Serena M. S. Dibárbora M. Olivera V. Metz G. E. Aspitia C. G. Pereda A.. (2021). Evidence of porcine circovirus type 2 and co-infection with ungulate protoparvovirus 1 (porcine parvovirus) in mummies and stillborn piglets in subclinically infected farm. Infect. Genet. Evol. 89:104735. doi: 10.1016/j.meegid.2021.104735, PMID: 33516972

[ref20] Sirisereewan C. Thanawongnuwech R. Kedkovid R. (2022). Current understanding of the pathogenesis of porcine Circovirus 3. Pathogens 11:64. doi: 10.3390/pathogens11010064, PMID: 35056012 PMC8778431

[ref21] Song C. Zhu C. Zhang C. Cui S. (2010). Detection of porcine parvovirus using a taqman-based real-time pcr with primers and probe designed for the NS1 gene. Virol. J. 7:353. doi: 10.1186/1743-422X-7-353, PMID: 21126330 PMC3014914

[ref22] Streck A. F. Truyen U. (2020). Porcine Parvovirus. Curr. Issues Mol. Biol. 37, 33–46. doi: 10.21775/cimb.037.033, PMID: 31822635

[ref23] Tu F. Zhang Y. Xu S. Yang X. Zhou L. Ge X.. (2021). Detection of pseudorabies virus with a real-time recombinase-aided amplification assay. Transbound. Emerg. Dis. 68, 2017–2027. doi: 10.1111/tbed.13849, PMID: 34273259

[ref24] Turlewicz-Podbielska H. Augustyniak A. Pomorska-Mól M. (2022). Novel porcine Circoviruses in view of lessons learned from porcine Circovirus type 2-epidemiology and threat to pigs and other species. Viruses 14:261. doi: 10.3390/v14020261, PMID: 35215854 PMC8877176

[ref25] VanderWaal K. Deen J. (2018). Global trends in infectious diseases of swine. Proc. Natl. Acad. Sci. U. S. A. 115, 11495–11500. doi: 10.1073/pnas.1806068115, PMID: 30348781 PMC6233110

[ref26] Vargas-Bermúdez D. S. Vargas-Pinto M. A. Mogollón J. D. Jaime J. (2021). Field infection of a gilt and its litter demonstrates vertical transmission and effect on reproductive failure caused by porcine circovirus type 3 (PCV3). BMC Vet. Res. 17:150. doi: 10.1186/s12917-021-02862-5, PMID: 33832500 PMC8028087

[ref27] Wang J. Zhang Y. Wang J. Liu L. Pang X. Yuan W. (2017). Development of a TaqMan-based real-time PCR assay for the specific detection of porcine circovirus 3. J. Virol. Methods 248, 177–180. doi: 10.1016/j.jviromet.2017.07.007, PMID: 28743583

[ref28] Wu X. Wang Z. Qiao D. Yuan Y. Han C. Yang N.. (2021). Porcine circovirus type 2 infection attenuates the K63-linked ubiquitination of STING to inhibit IFN-β induction via p38-MAPK pathway. Vet. Microbiol. 258:109098. doi: 10.1016/j.vetmic.2021.109098, PMID: 33984793

[ref29] Xu S. Chen D. Chen D. Hu Q. Zhou L. Ge X.. (2020). Pseudorabies virus infection inhibits stress granules formation via dephosphorylating eIF2α. Vet. Microbiol. 247:108786. doi: 10.1016/j.vetmic.2020.108786, PMID: 32768230

[ref30] Xu T. Zhang Y. H. Tian R. B. Hou C. Y. Li X. S. Zheng L. L.. (2021). Prevalence and genetic analysis of porcine circovirus type 2 (PCV2) and type 3 (PCV3) between 2018 and 2020 in Central China. Infect. Genet. Evol. 94:105016. doi: 10.1016/j.meegid.2021.105016, PMID: 34325052

[ref31] Zhang X. Shu X. Bai H. Li W. Li X. Wu C.. (2019). Effect of porcine circovirus type 2 on the severity of lung and brain damage in piglets infected with porcine pseudorabies virus. Vet. Microbiol. 237:108394. doi: 10.1016/j.vetmic.2019.108394, PMID: 31585642

[ref32] Zhao Y. Han H. Y. Fan L. Tian R. B. Cui J. T. Li J. Y.. (2019). Development of a TB green II-based duplex real-time fluorescence quantitative PCR assay for the simultaneous detection of porcine circovirus 2 and 3. Mol. Cell. Probes 45, 31–36. doi: 10.1016/j.mcp.2019.04.001, PMID: 30980890

[ref33] Zhou H. Pan Y. Liu M. Han Z. (2020). Prevalence of porcine Pseudorabies virus and its Coinfection rate in Heilongjiang Province in China from 2013 to 2018. Viral Immunol. 33, 550–554. doi: 10.1089/vim.2020.0025, PMID: 32397944

[ref34] Zhu J. H. Rawal G. Aljets E. Yim-Im W. Yang Y. L. Huang Y. W.. (2022). Development and clinical applications of a 5-Plex real-time RT-PCR for swine enteric coronaviruses. Viruses 14:1536. doi: 10.3390/v14071536, PMID: 35891517 PMC9324624

