# Supplementary material for: Establishment and application of multiplex real-time PCR for simultaneous detection of four viruses associated with porcine reproductive failure
Source: Front Microbiol. 2023 Feb 9;14:1092273. doi: 10.3389/fmicb.2023.1092273 (PMC9949525; doi:10.3389/fmicb.2023.1092273)
Supplement: Supplementary file 1 [file Table_1.DOCX]

**Supplementary Table S1. Primers for PRRSV, CSFV and PEDV**

| **Virus** | **Sequence (5′- 3′)** | | **Product size (bp)** |
| --- | --- | --- | --- |
| PRRSV | F | GAGTTTCAGCGGAACAATGG | 451 |
|  | R | GCCGTTGACCGTAGTGGAG |  |
| CSFV | F | AACATGGATGGTGTAACTGG | 343 |
|  | R | TTCTCTATAGTGTTGGTCATTCC |  |
| PEDV | F | GATATGTTTGTAATGGTAACTC | 503 |
|  | R | AGCATAGCTAAAAGGCAATGC |  |

**Supplementary Table S2. Clinical samples were tested in parallel by the multiplex real-time PCR, and the CT values from 43 samples co-infected with 2 or more pathogens are presented.**

| Region | Number | CT values from different fluorescent signals | | | |
| --- | --- | --- | --- | --- | --- |
|  |  | FAM (PCV2) | VIC (PCV3) | ROX (PPV) | Cy5 (PRV) |
| Changde | 14 | 23.29 | N/A | 22.98 | N/A |
|  |  | 21.65 | N/A | 25.93 | N/A |
|  |  | 23.08 | 24.35 | 23.01 | 29.57 |
|  |  | 11.09 | N/A | 14.80 | N/A |
|  |  | 23.27 | N/A | 20.29 | N/A |
|  |  | 33.51 | N/A | N/A | 20.29 |
|  |  | 30.68 | N/A | 28.83 | N/A |
|  |  | 26.03 | N/A | 31.12 | N/A |
|  |  | 33.32 | N/A | 29.99 | N/A |
|  |  | 27.93 | N/A | N/A | 18.96 |
|  |  | 27.40 | N/A | N/A | 20.18 |
|  |  | 26.32 | N/A | N/A | 17.46 |
|  |  | 27.11 | N/A | N/A | 19.49 |
|  |  | 27.90 | N/A | N/A | 18.01 |
| Yiyang | 10 | 27.64 | N/A | 28.69 | N/A |
|  |  | 22.29 | N/A | 20.21 | N/A |
|  |  | 25.41 | 26.89 | N/A | N/A |
|  |  | 30.00 | N/A | 27.50 | N/A |
|  |  | 28.68 | 25.86 | N/A | N/A |
|  |  | 11.99 | N/A | 13.93 | N/A |
|  |  | 15.03 | 25.29 | N/A | N/A |
|  |  | 14.89 | 22.43 | N/A | N/A |
|  |  | 16.35 | 23.22 | N/A | N/A |
|  |  | 17.00 | 22.35 | N/A | N/A |
| Hengyang | 6 | 22.56 | N/A | 25.99 | N/A |
|  |  | 21.37 | 23.76 | 22.72 | 28.27 |
|  |  | 22.64 | N/A | 23.64 | N/A |
|  |  | 17.21 | 19.72 | N/A | N/A |
|  |  | 14.57 | 20.79 | N/A | N/A |
|  |  | 15.68 | 24.83 | N/A | N/A |
| Zhuzhou | 3 | 26.45 | N/A | 26.71 | N/A |
|  |  | 14.30 | N/A | N/A | 20.92 |
|  |  | 14.11 | 18.32 | N/A | N/A |
| Huaihua | 2 | 19.98 | N/A | 22.97 | N/A |
|  |  | 32.83 | N/A | 21.25 | N/A |
| Yongzhou | 1 | 30.11 | N/A | 28.25 | N/A |
| Changsha | 3 | 14.95 | N/A | 16.86 | N/A |
|  |  | 22.75 | N/A | N/A | 16.24 |
|  |  | 28.22 | 24.13 | N/A | N/A |
| Xiangtang | 2 | 19.46 | 20.90 | 20.56 | 24.56 |
|  |  | 20.31 | N/A | 23.97 | 28.65 |
| Xiangxi | 1 | 32.20 | 25.60 | N/A | N/A |
| Liuyang | 1 | 23.25 | N/A | 24.56 | N/A |

**Supplementary Table S3. The positive detection rate of 10 copies and 1 copy standard plasmids at 20 times**

| **Virus** | **Two concentrations** | **Positive number** | **Positive rate** |
| --- | --- | --- | --- |
| PCV2 | 100 copies | 20 | 100% |
|  | 10 copies | 19 | 95% |
|  | 1copy | 13 | 65% |
| PCV3 | 100 copies | 20 | 100% |
|  | 10 copies | 19 | 95% |
|  | 1copy | 5 | 25% |
| PPV | 100 copies | 20 | 100% |
|  | 10 copies | 18 | 90% |
|  | 1copy | 5 | 25% |
| PRV | 100 copies | 20 | 100% |
|  | 10 copies | 17 | 85% |
|  | 1copy | 5 | 25% |
